# A 7-month-old girl with a suspected air embolism complication during a living-donor liver transplantation procedure: a case report

**DOI:** 10.3389/fped.2023.1271925

**Published:** 2023-11-14

**Authors:** Dan Huang, Liqun Yang, Weifeng Yu, Bo Qi

**Affiliations:** Department of Anesthesiology, Renji Hospital, Shanghai Jiaotong University, School of Medicine, Shanghai, China

**Keywords:** pediatric liver transplantation, cardiac events, perioperative management, congenital heart disease, case report

## Abstract

**Background:**

Pediatric liver transplantation is an important modality for treating biliary atresia. The overall survival rate of pediatric liver transplantation has significantly improved. The incidence of perioperative cardiac events was evaluated, and risk factors were also investigated in adult patients undergoing liver transplantation in previous studies. To the best of our knowledge, this is the first case of a cardiac event during a pediatric living-donor liver transplantation.

**Case summary:**

Our report describes the management of cardiac events during a liver transplantation in a 7-month-old girl. The ST segment began to increase to 3.0 mm immediately after reperfusion, with peak ST-segment elevation reaching 13.2 mm after 45 min. The procedure ended uneventfully after continuous symptomatic and etiological treatment. It was considered to be the occurrence of an acute air embolism complication during the procedure based on the electrocardiograph and biomarkers. An echocardiogram during follow-up showed a patent foramen ovale with a left-to-right shunt tract width of 2.7 mm.

**Discussion:**

Pediatric liver transplantation has become a state-of-the-art treatment for children with end-stage liver disease and can improve the quality of life to some extent. These children may be complicated with congenital heart disease, which increases the risk of surgery. Application of echocardiogram, close monitoring, and appropriate management may reduce the incidence of perioperative cardiac events.

## Introduction

Pediatric liver transplantation is an important modality for treating biliary atresia. Living-donor liver transplantation has been widely performed to solve the problem of donor shortage (1). The overall survival rate of pediatric liver transplantation has significantly improved, while 1-year all-cause mortality is 4.78%, remaining unsatisfactory (2). Incidence of perioperative major cardiac events associated with late mortality was reported to be 2.6%–12.3% in adult patients undergoing liver transplantation (3–5). Despite perioperative cardiac events in pediatric living-donor liver transplantation being rarely reported in previous studies, the risk of death from acute air embolism complications during the procedure, a relatively infrequent event but associated with significant morbidity and mortality, can never be eliminated (6). Although the frequency of this complication in pediatric liver transplantation has not been mentioned yet, iatrogenic air embolism complicates 2.65 in 100,000 hospitalizations proposedly and the true incidence may be greater since many air embolisms are asymptomatic (7). We report our experience with acute air embolism complications during the procedure after reperfusion.

## Case presentation

A 9.3-kg 7-month-old girl with congenital biliary atresia presented for living-donor liver transplantation. At 6 months of age, she developed hepatic dysfunction and was treated with symptomatic therapy. Her preoperative hemoglobin (Hb) was 77 g/L, albumin was 30.7 g/L, total bilirubin was 430.4 μmol/L, and prothrombin time was 17.3 s, with no electrocardiograph (ECG) abnormality or prominent heart murmur. Her preoperative blood pressure was 90/50 mmHg and the heart rate was 130 bpm.

On arrival in the operating room, ECG leads and a pulse oximeter were placed and continuously monitored. General anesthesia was induced by inhalation of sevoflurane 8% (vol) with transvenous midazolam 1 mg, sufentanyl 5 μg, and rocuronium 10 mg, followed by intubation with an endotracheal tube. Subsequently, a 24G left radial arterial catheter was inserted for continuous invasive arterial blood pressure (IABP) monitoring. A 4F double-lumen intravenous catheter was placed in the right internal jugular vein for continuous central venous pressure (CVP) monitoring. Anesthesia was maintained with expiratory sevoflurane 2% (vol), sufentanyl 10 μg/h, and rocuronium 5 mg/h. Arterial blood gas values after intubation were pH 7.300, arterial oxygen pressure (PaO_2_) 154 mmHg, Hb 5.5 g/L, and potassium 2.6 mmol/L during intermittent positive-pressure ventilation with a fraction of inspired oxygen 0.6. The patient received 20% human serum albumin 50 ml and red blood cells 1U. Vital signs were stable at 25 min of the hepatic-free stage, and arterial blood gas values 20 min after portal occlusion were pH 7.310, base excess −6.8 mmol/L, Hb 8.8 g/L, and potassium 3.4 mmol/L, while core body temperature was maintained at 37°C. She received 5% sodium bicarbonate 30 ml.

**Figure 1:**
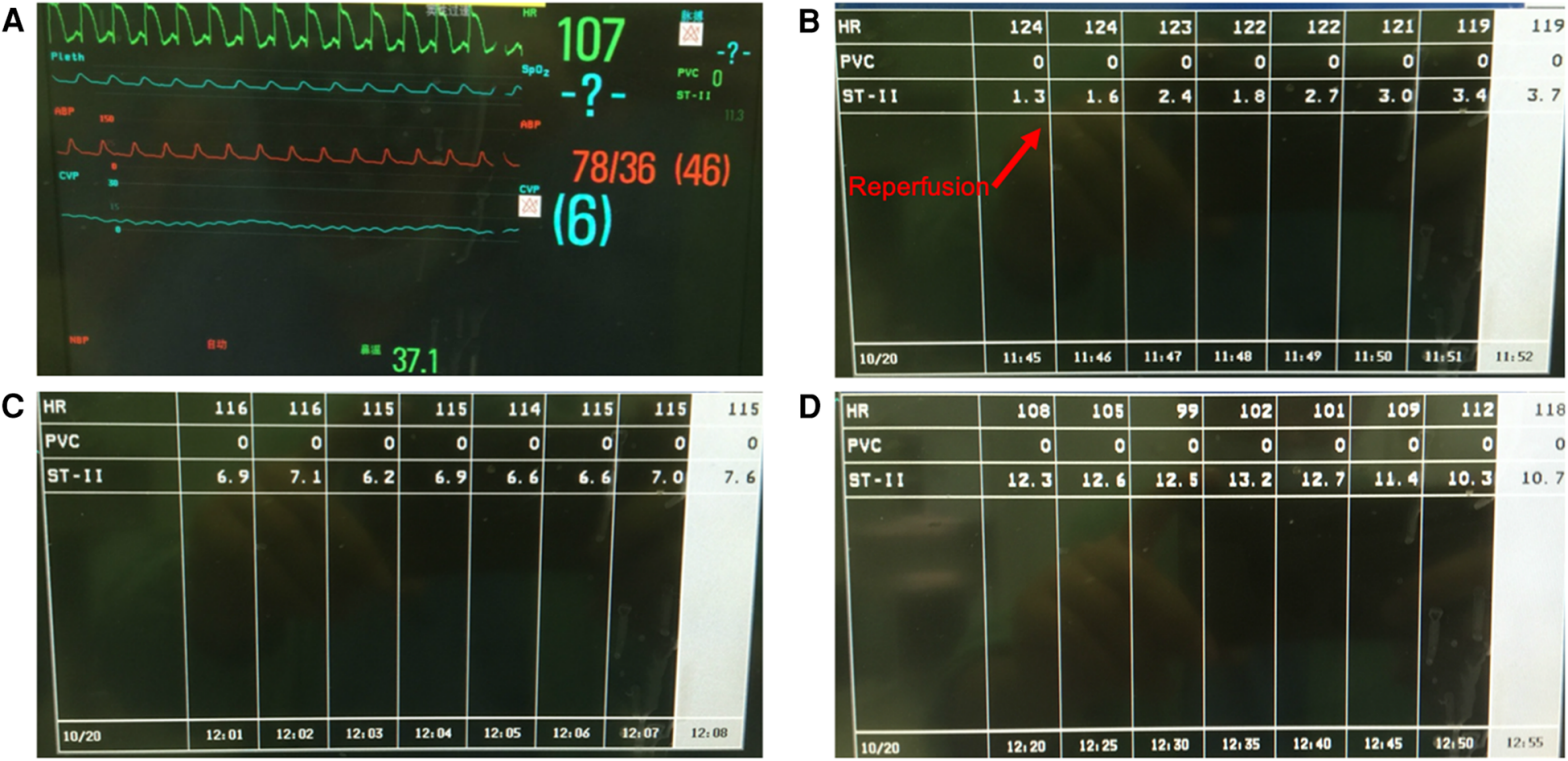
(**A**) The patient's vital signs during ST-segment elevation on the monitor display. (**B**) Immediately after reperfusion, the ST segment increased to 3.7 mm within 7 min. (**C**) The ST segment gradually increased. (**D**) ST-segment elevation reached 13.2 mm 50 min after reperfusion.

Immediately after reperfusion, IABP, especially systolic blood pressure, steeply decreased to 64/45 mmHg, followed by a heart rate decrease to 117 bpm. IABP quickly returned to 80/50 mmHg without treatment. However, the ST segment began to increase to 3.0 mm and gradually reached 13.2 mm within 45 min (Figure 1). The patient's blood pressure (BP), heart rate (HR), and SpO_2_ were in the normal range during this period.

**Figure 2:**
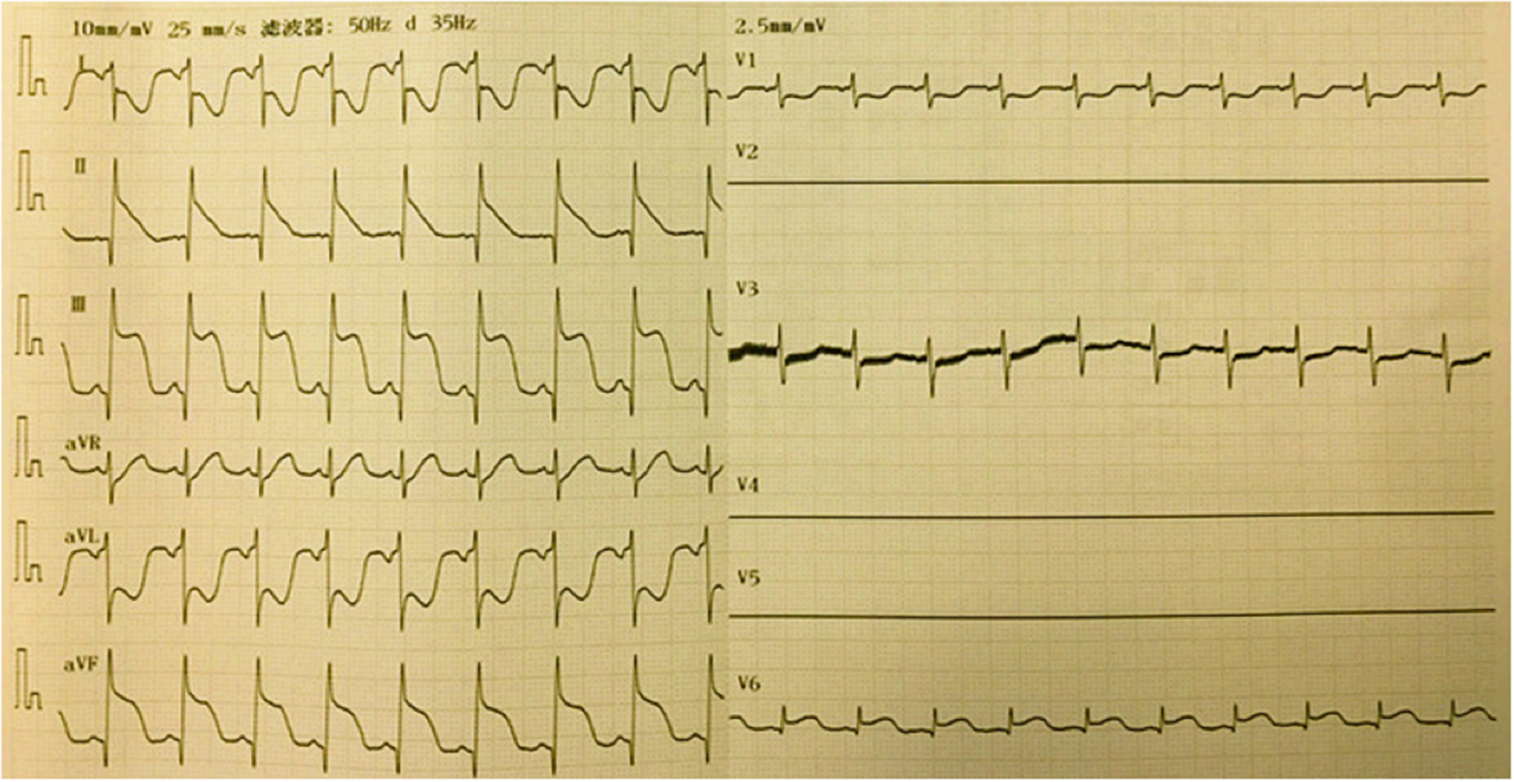
A full-lead ECG was monitored at the surgical bedside, showing ST-segment elevation in II, III and aVF leads, and ST-segment depression in I and aVL leads.

**Figure 3:**
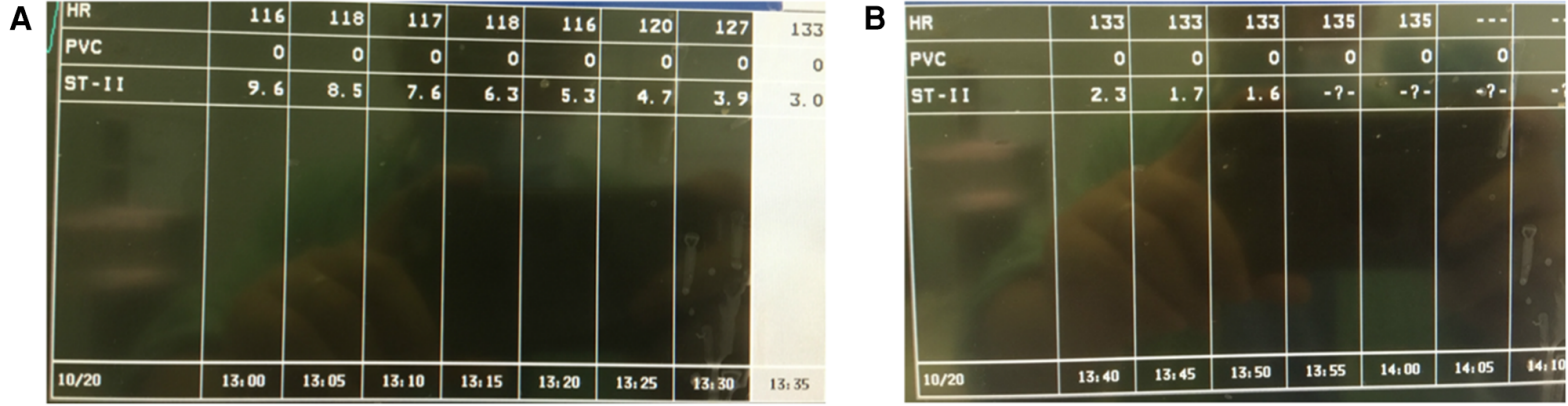
Nitroglycerin infusion at a dosage of 2 μg/kg/min and potassium chloride at a dosage of 0.5 mg/kg/min were applied. (**A**) ST-segment elevation gradually reduced to 3.9 mm 100 min later. (**B**) ST-segment elevation gradually reduced to 1.6 mm 2 h later.

For further diagnostic workup, the respiratory circuit, tracheal tube, and anesthesia machine were also checked as soon as possible to confirm that all processes were normal. A full-lead ECG was monitored at the surgical bedside, showing the ST-segment elevation (STE) in II, III, and Augmented Voltage Foot (EKG lead) (aVF) leads, and ST-segment depression in I and Augmented Voltage Left Arm (EKG lead) (aVL) leads, consistent with subendocardial and inferior subepicardial myocardial injuries (Figure 2). Arterial blood gas was detected, and values were in normal range except for potassium 3.1 mmol/L. Myocardial infarction markers were also detected, which showed that cardiac troponin (cTnl), creatine kinase-MB (CK-MB), and myoglobin (MYO) had all increased to more than 2 times the normal values. After 2 h of nitroglycerin infusion at a dosage of 2 μg/kg/min and potassium chloride at a dosage of 0.5 mg/kg/min, STE gradually reduced to 1.6 mm (Figure 3).

**Figure 4:**
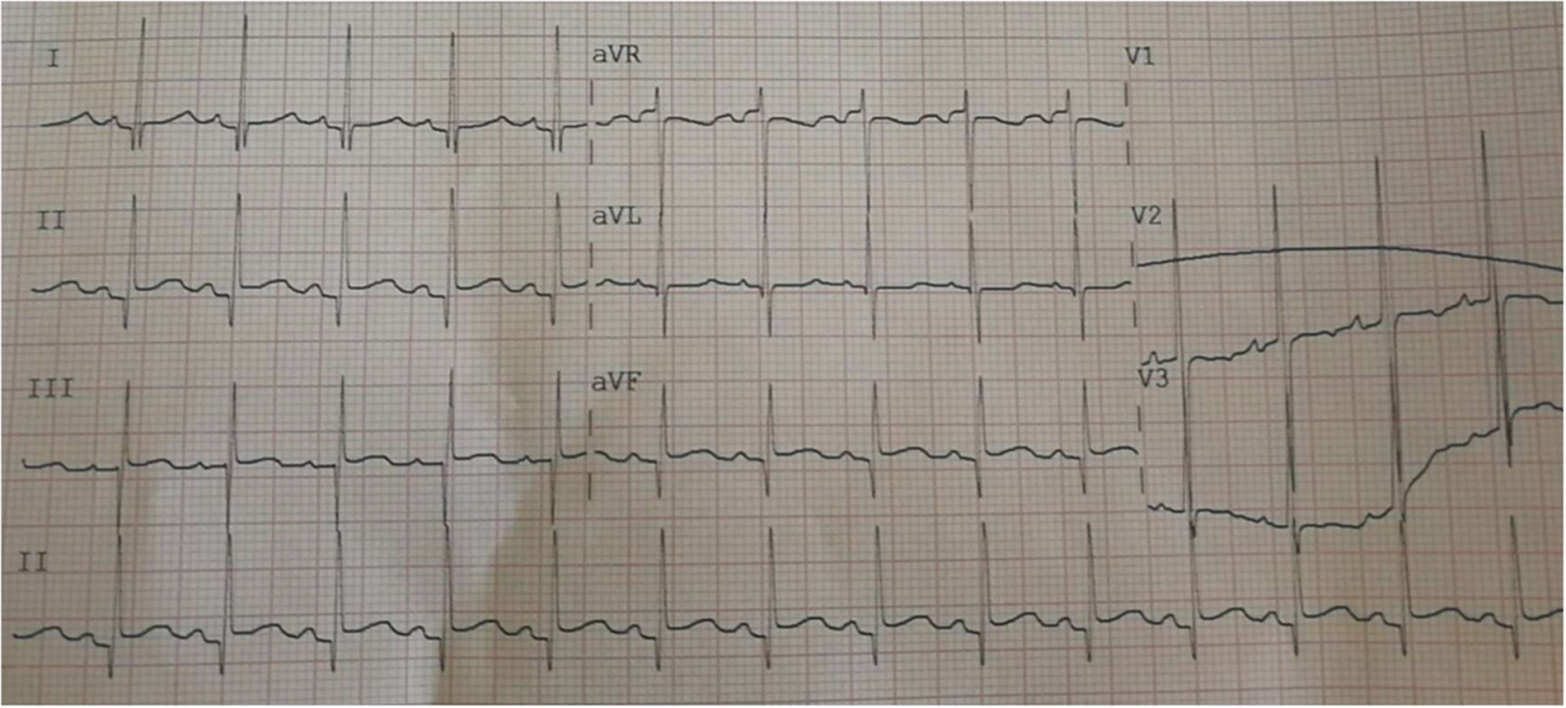
A full-lead ECG was monitored immediately after admission to the transplantation intensive care unit, showing ST-segment slight elevation in II, III, and aVF leads.

The procedure was completed 3 h after reperfusion, with consistently stable vital signs. A full-lead ECG was monitored immediately after admission to the transplantation intensive care unit, showing slight ST-segment elevation in II, III, and aVF leads (Figure 4). Markers of myocardial infarction gradually decreased to almost normal levels during the first few days after the procedure (Supplementary Figure S1). The patient was successfully discharged from the hospital 12 days after surgery. An echocardiogram showed a patent foramen ovale with a left-to-right shunt tract width of 2.7 mm. No sequela related to air embolism was identified postoperatively.

## Discussion

This report describes a rare acute air embolism complication during a liver transplantation procedure in a young infant. The procedure ended uneventfully after continuous symptomatic and etiological treatment.

Among pediatric patients undergoing liver transplantation, those with familial hypercholesterolemia associated with severe atherosclerosis in childhood lead to death from myocardial infarction before the age of 20 years (8). An infant with fulminant hepatic failure was reported to succumb to a myocardial infarction 24 h following her transplant with good graft function, and no cause for her infarct was detected at autopsy (9). To date, no air embolism complication during a living-donor liver transplantation procedure has been reported in pediatric patients with biliary atresia. However, congenital heart disease and biliary atresia were unexpected prevalent associations in several cohorts (10–12). A contemporary cohort study demonstrated liver transplantation recipients with biliary atresia may have an associated diagnosis of congenital heart disease at a rate of 16.4% (13). An echocardiogram after discharge showed a patent foramen ovale with a left-to-right shunt tract width of 2.7 mm in this case, indicating the air embolism complication during the procedure after reperfusion was probably related to congenital heart disease. As long as the shunt is from left to right, the presence of embolisms in the coronary, cerebral, or systemic circulation is unlikely; however, when closure of the foramen ovale does not occur and it remains patent, it can favor a right-to-left shunt during the pressure crossing that occurs in the respiratory cycle, mainly in end-diastole, or in situations in which pressure in the right atrium increases (e.g., cough or the Valsalva maneuver). A transient increase in right heart pressure during reperfusion may have induced a right-to-left shunt in the current case. It is considered that the air entered the coronary artery through the open foramen ovale from the inferior vena cava during the anastomosis of the liver graft, resulting in an embolism.

An acute air embolism complication during the procedure was diagnosed based on the electrocardiograph and biomarkers in this case. Accurate upper reference limits for defining myocardial injury using high-sensitivity cTnl were provided based on established normative data from a representative pediatric sample among participants of 1–18 years of age in the 1999–2004 National Health and Nutrition Examination Survey, and 6 ng/L as the normative upper reference limit was recommended in female children (14). The peak cTnl of the girl in our report was 7.41 ng/ml, more than 1,000 times the reference limit, at the end of operation. Additionally, transesophageal echocardiography (TEE) is likely to be more helpful in diagnosing an air embolism, revealing the amount of air transiting the right side of the heart and a stream of bubbles entering the left heart, also facilitating image-guided aspiration of trapped air via the center catheter (15). However, placement of TEE was almost impossible due to space, equipment, and pediatric anatomy at that moment. Therefore, continuous ECG monitoring and rapid diagnosis followed by intervention is crucial in the management of this emergency.

Recent guidelines for acute coronary syndrome recommend administration of nitroglycerin (16). Timely detection and 2 h of nitroglycerin infusion may have played a role in the successful experience in this case. Therefore, it is important to carefully monitor ST segment changes on ECG after reperfusion in living-donor liver transplantation. The detection and prompt treatment of embolisms are necessary. We advocate the application of echocardiograms before surgery to be effective for screening congenital heart disease in liver transplantation recipients. Thankfully, this patient did not suffer any long-term consequences of the embolism.

## Data Availability

The original contributions presented in the study are included in the article/**Supplementary Material**, further inquiries can be directed to the corresponding author.

## Supplementary material

Supplementary Figure S1 Markers of myocardial infarction gradually decreased to almost normal levels during the first few days after the procedure. cTnl, cardiac troponin; CK-MB, creatine kinase-MB; MYO, myoglobin; STE, ST-segment elevation; EO, end of operation; POD, post-operative day.
