# Supplementary material for: A 7-month-old girl with a suspected air embolism complication during a living-donor liver transplantation procedure: a case report
Source: Front Pediatr. 2023 Nov 14;11:1271925. doi: 10.3389/fped.2023.1271925 (PMC10682432; doi:10.3389/fped.2023.1271925)
Supplement: Supplementary file 1 [file Table1.docx]

**Timeline**

| Time point | Event |
| --- | --- |
| 9:30 October 20 2022  (operation onset) | Patient underwent a living donor liver transplantation, due to congenital biliary atresia. |
| 11:20 October 20 2022  (portal occlusion) | Vital signs were stable at 25 min of hepatic-free stage. |
| 11:45 October 20 2022  (reperfusion) | Immediately after reperfusion, IABP, especially systolic blood pressure, steeply decreased to 64/45 mmHg, followed by heart rate increase to 117 bpm. IABP quickly returned to 80/50 mmHg without treatment. |
| 11:50 October 20 2022  (ST-segment elevation onset) | ST-segment began to increase to 3.0 mm. |
| 12:35 October 20 2022  (peak ST-segment elevation) | ST-segment gradually reached 13.2 mm within 45 min. |
| 13:50 October 20 2022  (end of operation) | The procedure was completed 3 h after reperfusion, with consistently stable vital signs. |
| October 26 2022  (transferred to a general ward) | Patient recovered, no additional monitoring required. |
| November 2 2022  (discharge from the hospital) | Patient was successfully discharged from the hospital 12 days after surgery. |
| November 9 2022 (follow-up) | An echocardiogram showed a patent foramen ovale with a left-to-right shunt tract width of 2.7 mm. |
