# Supplementary figures and images for: A 7-month-old girl with a suspected air embolism complication during a living-donor liver transplantation procedure: a case report
Source: Front Pediatr. 2023 Nov 14;11:1271925. doi: 10.3389/fped.2023.1271925 (PMC10682432; doi:10.3389/fped.2023.1271925)

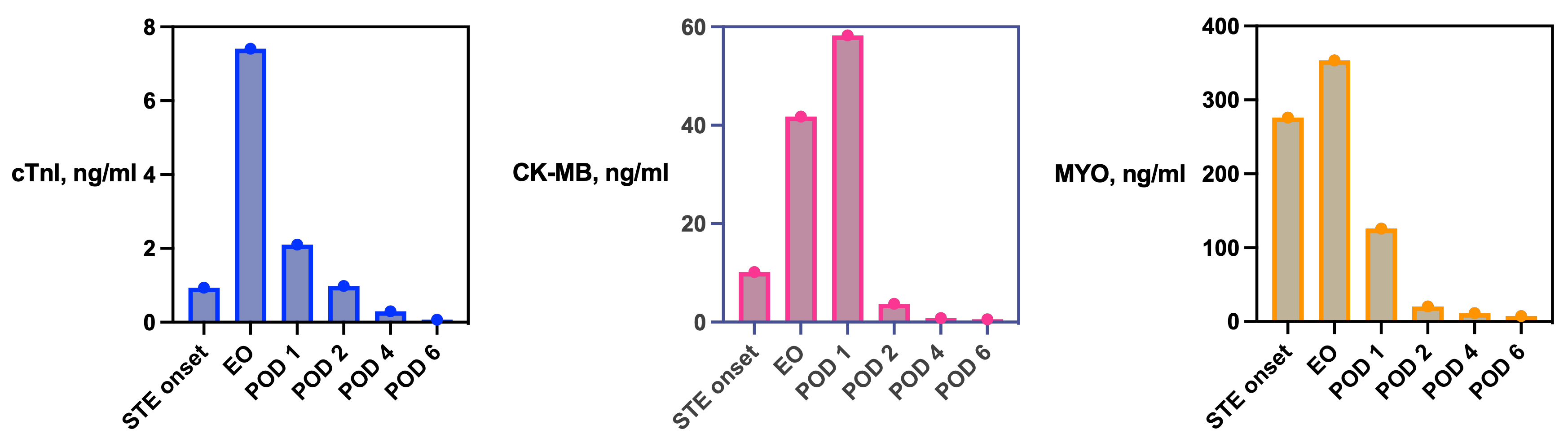

Supplement: Supplementary Figure S1 — Markers of myocardial infarction gradually decreased to almost normal levels during the first few days after the procedure. cTnl, cardiac troponin; CK-MB, creatine kinase-MB; MYO, myoglobin; STE, ST-segment elevation; EO, end of operation; POD, post-operative day. [file Image1.tiff]
